# Laparoscopic assisted repair of strangulated obturator hernia—Way to go

**DOI:** 10.1016/j.ijscr.2019.07.029

**Published:** 2019-07-23

**Authors:** Casey Joe, Vinayak Gowda, Suman Koganti

**Affiliations:** Department of Surgery, Bronx-Care Health System, Icahn School of Medicine at Mount Sinai, Milstein 4A, 1650 Selwyn Avenue, New York, 10457, USA

**Keywords:** Obturator hernia, Surgery, Laparoscopy, Mesh

## Abstract

**Introduction:**

Obturator hernias are a rare subtype of intra-abdominal hernias that present with considerable challenges in both diagnosis and surgical repair. Delays in diagnosis and management significantly increases the Morbidity and Mortality.

**Presentation of case:**

An elderly 70-year-old female presented with Lower abdominal pain radiating to the medial side of the left leg over a year that has become persistent and continuous over the 3 day period. Patient was found to have a Richter's type of strangulated obturator hernia on diagnostic laparoscopy. A laparoscopic assisted resection of the strangulated small bowel and repair of the Hernia was performed. Postoperatively patient had an uneventful recovery.

**Discussion:**

Surgical treatment is the standard of care for an obturator Hernia. Increasing number of laparoscopic repairs are being performed in the recent past. Minilaparotomy greatly facilitates the resection and anastomosis of a dead bowel without jeopardizing cosmesis and mesh placement at the time of repair.

**Conclusion:**

Heightened awareness of the condition combined with our proposition of a Hybrid repair achieves optimal results in an emergency setting. A Laparoscopic assisted repair of a strangulated hernia provides a durable repair and at the same time offers the patient benefits of minimally invasive surgery.

## Highlights

• Heightened awareness and understanding of Obturator Hernias is important.
• Delays in diagnosis and treatment increase Morbidity and Mortality.
• Laparoscopic techniques of repair are met with increasing success in both elective and an emergency setting.

## 1 Introduction

Obturator hernias are a rare type of pelvic hernia with an incidence of 0.07–1% of all intra-abdominal hernias and the cause of 0.2% to 1.6% of all small bowel obstruction. They occur more commonly in thin, elderly females between the ages of 70–90 and with a higher prevalence in Asian countries [[1], [2], [3], [4]]. Obturator hernias often present as an acute onset of bowel obstruction with strangulation and incarceration. Most commonly, obturator hernias are seen on the right side since the left sided obturator foramen typically covered by sigmoid colon [3,4]. We describe an elderly lady who presents with a Richter’s type of obturator hernia that was successfully repaired laparoscopically and made an uneventful recovery. This work is in compliance with the SCARE guidelines [5].

## 2 Case presentation

**Fig. 1:**
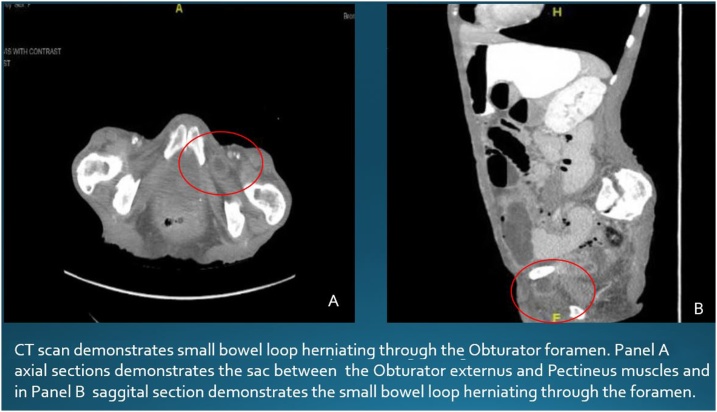
CT scan demonstrates small bowel loop herniating through the Obturator foramen. Panel A axial sections demonstrates the sac between the Obturator externus and Pectineus muscles and in Panel B saggital section demonstrates the small bowel loop herniating through the foramen.

**Fig. 2:**
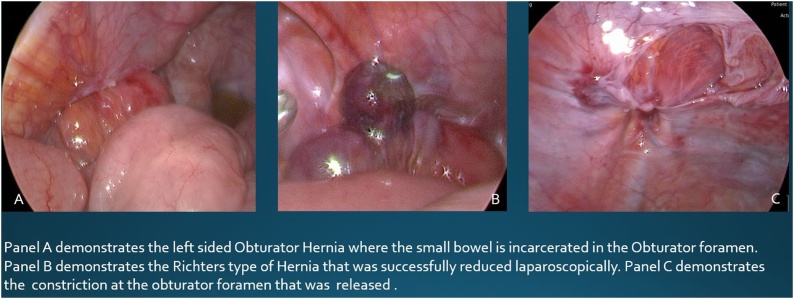
Panel A demonstrates the left sided Obturator Hernia where the small bowel is incarcerated in the Obturator foramen. Panel B demonstrates the Richters type of Hernia that was successfully reduced laparoscopically. Panel C demonstrates the constriction at the obturator foramen that was released.

A 70 year old female with chronic obstructive pulmonary disease (COPD) on home oxygen with no history of previous surgeries presented with lower abdominal pain for a year which has become constant and severe for 2 days. She had nausea, vomiting and was not passing flatus for 3 days. On physical exam vital signs were within normal limits. Abdomen was mildly tender in the suprapubic region with no peritoneal signs. There are no palpable hernial swellings or lymph nodes. On adduction and medial rotation of left thigh, patient complained of increased pain in her medial left leg. Laboratory test showed increased lactic acid level. Abdominal CT scan (Fig. 1) was done which showed dilated small bowel loops and findings suspicious for incarcerated small bowel containing left obturator hernia. Patient refused nasogastric tube gastric decompression. Patient was taken to the operating room for surgical management of the incarcerated hernia. In the operating room, after achieving penumoperitoneum, a 5 mm 30 ° laparoscope was introduced and dilated small bowel was noted with free peritoneal fluid. Two 5 mm port was placed on each side of the abdomen. After displacing distended small bowel loops, a loop of small bowel herniating into the left obturator canal was observed. Attempt to reduce left obturator hernia by gentle traction was successful (Fig. 2). A Richter’s type (partial enterocele with protrusion or strangulation of only part of the circumference of the intestine's anti mesenteric border) of Obturator hernia was noted and gangrenous portion of approximately 3 cm of small bowel was noted.

A small transverse pfannensteil incision (5 cm) was made just above the pubic symphysis and the loop of gangrenous small bowel was delivered out and resected with a GIA stapler. After that peritoneum and the fascia was closed and pneumoperitoneum recreated. Subsequently a Vicryl woven mesh cut to appropriate size was tacked around the left obturator canal to buttress the hernial defect. The neurovascular bundle and the obturator foramen was clearly visible laparoscopically and was protected. No entrapment of bowel or omentum was observed at the end. Pneumoperitoneum was desufflated and ports were removed. Post operatively patient made an uneventful recovery.

## 3 Discussion

Obturator hernias were first described by Arnaud de Ronsil in 1724 and the first successful surgical repair of these was performed by Henry Obre in 1851 [4]. Obturator hernias are associated with significant delays in diagnosis and management because of their uncommon occurrence and because of the absence of a palpable mass on physical exam [[2], [3], [4]]. Concomitant conditions such as chronic constipation, chronic obstructive pulmonary disease, ascites, and kyphoscoliosis also predispose patients to herniation by increasing the intra-abdominal pressure and relaxing the peritoneum [2]. Poor general condition of the patient and associated delays in diagnosis and management lead to a heightened morbidity and mortality [[1], [2], [3], [4],^6^]

## 4 Surgical anatomy

The obturator foramen which is the largest foramen in the body is formed by the pubis and ischium and is covered by an obturator membrane anterosuperiorly. The foramen is closed by a strong quadrilamellar musculoaponeurotic barrier consisting of an internal and an external obturator membrane and an internal and an external obturator muscle. The membranes are joined below and separated above and are encased by the muscles on both sides. The obturator canal is situated in the large cranial part of the foramen. The obturator canal is approximately 2 to 3 cm long, running obliquely downward and upward. The obturator nerve and blood vessels pass through this canal and are covered peripherally by fatty tissue. Usually this fatty tissue helps fills the space in the obturator canal, thus, decreasing the risk for protrusion of small bowel. With severe cachexia and/or malnutrition, there is loss of this fatty tissue which can increase the risk for an obturator hernia to develop [7,8,9]. Three different types of OH are described based on the relationship of the Hernia to the muscle layers covering the Obturator foramen [4]. When the hernia is approached from thigh knowing the type of hernia is important as it can cause difficulty in finding the sac [2,4].

## 5 Clinical presentation

The eponym “**Hernia of a skinny old lady**” underscores the phenotype of the Obturator Hernia, seen in the elderly and emaciated women. The hernia has a female predilection because they have a broader pelvis with a larger triangular obturator canal opening with a greater transverse diameter. Prior pregnancy is cited as a common risk factor [2,4,9]. OH most commonly presents as intestinal obstruction in an elderly lady with no prior operations [10]. Three signs Obturator neuralgia, Howship-Romberg sign, Hannington –Kiff sign (absent adductor reflex in the thigh in the presence of a positive patellar reflex due to compression of obturator nerve) are described as specific for a strangulated OH [4]. Of these the Howship –Romberg sign which is pain in the medial thigh caused by compression of the anterior division of obturator nerve when the extremity is extended, adducted and internal rotated is considered pathognomonic but seen only 15–50% of the time [2,4]. Patients with advanced gangrene and delayed presentation can present with thigh sepsis [11]. Ultrasound, CT, MRI are all useful in imaging an OH. CT scan in both an acute and non-emergent setting is highly specific for the diagnosis with accuracy above 90%. CT is also useful in defining the relation of the sac to the muscles overlying the obturator foramen and identifies the type of Hernia which is important when approaching the Hernia from the Groin.

## 6 Management

Surgical repair of the Hernia is the standard of care for Obturator Hernia. Several open and laparoscopic techniques are described in the literature to repair the Hernia [10,12]. As the incidence of Hernia is low, there are no large scale data to demonstrate the superiority of one over the other. The choice of repair depends on the acuity of presentation, experience of the surgeon and expertise in laparoscopic surgery.

Conventionally open approaches are used. Four different types Abdominal, retropubic, obturator, and inguinal approaches are described [13]. In an emergency setting, the abdominal approach via a low midline incision is most commonly favoured, as it allows adequate exposure of the obturator ring as well as the identification and resection of any ischaemic bowel. Closure of the defect by synthetic mesh is not advocated in the setting of perforation or gangrenous bowel. In such cases, simple closure with a non absorbable suture leaving the sac in situ is most appropriate and has an acceptable recurrence rate of less than 10% [12,14].

Recently Laparoscopic techniques (Both transabdominal and Extra-peritoneal) are increasingly being used for the repair of OH [12,[15], [16], [17]]. As familiarity with TAPP and TEPP procedures for groin hernias improved, surgeons have become more and more facile with the laparoscopic groin hernia repairs which seem to have influenced the trend of increasing Laparoscopic repairs being performed. Reported benefits include excellent visualiasation and acess to the Hernia versus the difficulty of retraction and poor visibility of the Hernial orifice especially in a narrow pelvis [12]. A transperitoneal approach like the one described in our case is favoured in an emergency setting. It allows for inspection of the entire bowel, reduction of the incarcerated Hernia and resection anastomosis of the bowel if viability is compromised. The key principle of repair (be it open or laparoscopic) in the management of Incarcerated OH is to release the constriction at the neck at the Hernial orifice and reduce the Hernial contents. This could be performed laparoscopically with a peanut or a right angle and divide the constriction with an endoshears in a postero-medial direction with care taken to avoid injury to the obturator vessels [4,12]. A mini laparotomy greatly facilitates the repair of a strangulated bowel and conserves time in an emergency setting. Lastly the use of a prosthetic mesh in the setting of a potentially contaminated field is undesirable and generally not advocated. However there are reports of successful repair with a mesh in the setting of a strangulated bowel [18]. If the extent of gangrene is limited (Richter’s type described above) and there is no spillage of enteric content, a case can be made for the use of a prosthetic mesh at the time of Hernia repair.

## 7 Conclusion

Awareness of the pathophysiology of Obturator Hernia is important. Delays in diagnosis and treatment can significantly heighten the morbidity and mortality. Rarity of the condition and the absence of telltale clinical signs imposes challenges in early diagnosis and treatment. However a heightened awareness of the condition, dedicated cross sectional imaging and an early operative repair provides the best long term outcomes.

## Declaration of Competing Interest

No conflicts of interest.

## Sources of funding

All the authors report no financial acknowledgments involved for this case report and declare no potential conflicts of interest.

## Ethical approval

Study exempt from ethical approval.

## Consent

Written informed consent was obtained from the patient for publication of this case report and accompanying image.

## Authors contribution

Study concept and design: Suman B Koganti MD.

Data collection: Casey Joe MD.

Data analysis and interpretation: Suman B Koganti MD, Vinayak Gowda MD.

Writing the paper: Casey Joe MD, Suman B Koganti MD.

## Registration of Research Studies

None.

## Guarantor

Vinayak Gowda MD.

## Provenance and peer review

Not commissioned, externally peer-reviewed.
